# “Overconfidence” versus “helplessness”: A qualitative study on abstinence self-efficacy of drug users in a male compulsory drug detention center in China

**DOI:** 10.1186/s13011-016-0073-2

**Published:** 2016-08-31

**Authors:** Yan Zhang, Bing Feng, Wenxiu Geng, Laurence Owens, Juzhe Xi

**Affiliations:** 1 School of Psychology and Cognitive Science, East China Normal University, Shanghai, China; 2 School of Education, Flinders University, Adelaide, Australia; 3 Social Science Division, University of Chicago, Chicago, USA; 4 School of Basic Medical Science of Shutcm, Shanghai TCM University, Shanghai, China

**Keywords:** Drug use, Abstinence self-efficacy, Compulsory drug detention, Drug policy, Learned helplessness, Attribution theory, Qualitative method

## Abstract

**Background:**

Compulsory drug detention is the most frequent way to control drug use in China; however, it has often been criticized. This qualitative study aimed to investigate abstinence self-efficacy and its sources of drug users in a compulsory male drug detention center in Shanghai, China, and the attitudes of the drug users to this form of rehabilitation.

**Methods:**

Thirty-six participants were interviewed (semi-structured, in depth) about their history of drug use and rehabilitation, self-evaluation of addiction, motivations to abstain, plans for the future and attitudes toward rehabilitation. A thematic analysis was undertaken of the transcripts with responses to interview questions being coded for content.

**Results:**

Two main types of self-efficacy were found – “overconfidence” (*n* = 16) and “helplessness” (*n* = 17). Overconfident participants underestimated their levels of addiction, overestimated their self-control and held external motivations and attributions. In contrast, helpless participants overestimated their levels of addiction, underestimated their self-control and had internal motivations and attributions. Compared to overconfident participants, helpless participants had more relapse history, and were more inclined to interpret relapse as a failure and attribute relapse to themselves. More helpless participants were abandoned by their family members, and received blame from the family members instead of encouragement, but their family members motivated them to abstain. Helpless participants experienced more negative emotions and had worse physical status. They said compulsory detention was a strong support for them and was the most effective way to abstain; while overconfident participants said compulsory detention was not necessary and not useful.

**Conclusion:**

It is important to increase the motivation of overconfident drug users and the perceived control of helpless drug users. Compulsory drug detention has strengths in supporting drug users who feel helpless to resist drug use. Adjustments and improvements of compulsory drug detention are suggested.

**Electronic supplementary material:**

The online version of this article (doi:10.1186/s13011-016-0073-2) contains supplementary material, which is available to authorized users.

## Background

### Drug control and compulsory detention in China

By the end of 2012, the total number of Chinese drug users in rehabilitation was 2.1 million, of which 60.6 % used opioid drugs (such as heroin) and 38 % used synthetic drugs (such as ice (methamphetamine)) [1]. And over 63 % of drug users relapsed within 3 years.

On June 1st, 2008, the new *Narcotics Control Law of the People’s Republic of China* was adopted by the 10th National People’s Congress of China [2]. The law defined clearly four methods of rehabilitation: voluntary drug rehabilitation, community drug rehabilitation, compulsory drug detention and community recovery [2, 3].

**Fig. 1:**
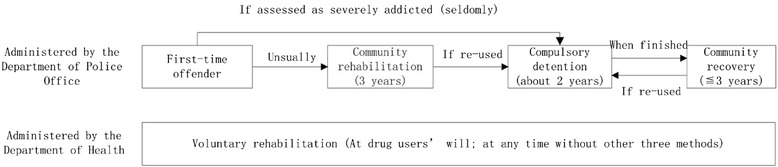
Relationships between four drug-rehabilitation methods in China

According to the law, the assessment of drug addiction is determined by the Administrative Department of Health, the Drug Supervision and Administration Department and the Department of Public Security under the State Council [2]. The first-time drug offender is required to take a three-year community drug-rehabilitation treatment. During or after community drug-rehabilitation, if a person re-uses drugs, he or she is forced to take the compulsory drug detention (duration varies from 1 to 3 years, but usually 2 years). After the compulsory drug detention, he or she may need to take the community recovery (duration varies in different provinces, but should be no more than 3 years). At other times, drug users could take voluntary rehabilitation of their own accord; or their family members could send them to this. The relationships between the four methods of rehabilitation in China are illustrated in Fig. 1 (For more information, see [2–5]).

Drug users or their family members need to pay for access to voluntary rehabilitation centers, but they could leave at any time. The centers are administered by the Department of Health and are usually built in public or private hospitals, where methadone administration is the main treatment. The other three methods are free and administered by the Department of Public Security. During community rehabilitation and community recovery, drug users rehabilitate at home. They could live normal lives, but are required to take urine tests and attend drug education classes twice per month, monthly or quarterly (depending upon the addiction assessment). Some communities may provide additional treatments, such as psychological counselling.

During compulsory detention, drug users rehabilitate in a special and closed environment with other drug users, separated from family and work. Its compulsory detention character makes it distinct from the other three methods and it has been considered as a violation of human rights [6–8]. However, in compulsory detention centers drug users receive treatments regularly, even though treatments vary across centers [6] and the treatment efficacy has been frequently questioned [7, 9]. Moreover, there is evidence showing that mandatory treatment can produce positive outcomes [10].

Compulsory drug detention is the most frequent way to control drug use in China and some Asian countries [11–14]. By the end of 2012, more than three hundred thousand people were given compulsory drug detention in 678 compulsory drug detention centers across China [1].

The term of compulsory drug detention is usually 2 years, although the duration could vary from 1 to 3 years, according to the severity of addiction and drug users’ progress in centers. The evaluation of progress is largely based on 1) the outcome of the detainee’s productive labor, 2) the grade on their drug education test subsequent to their drug education classes and 3) the absence of physical attacks upon or conflicts with others [2, 3].

Although compulsory drug detention generally lasts for approximately 2 years, the relapse rate within 1 year of completing compulsory rehabilitation, ranged from 85 % [15] to 98.9 % [16]. Because of these large relapse rates, researchers have questioned its effectiveness [6, 7, 17]. Apart from investigating the efficacy of treatment, some researchers also tried to find out the reasons for relapse from drug users themselves, particularly relating to self-efficacy as a causal factor [16, 18]. In this study, we set out to examine the self-efficacy of drug users in a compulsory detention center.

### Self-efficacy and drug use

Self-efficacy, first proposed by Bandura, is the confidence in being able to change a behavior [19, 20]. Bandura argued that self-efficacy varies along three dimensions: strength, magnitude and generality, implying that self-efficacy must be viewed as situation dependent [21]. Some researchers also pointed out that self-efficacy varies across time and situation [22–24]. We focus only on specific abstinence self-efficacy of drug users during compulsory detention.

Abstinence self-efficacy is the confidence in one's ability to effectively engage in behaviors to maintain substance use abstinence [25]. Researchers found that abstinence self-efficacy is a predictor, even a sole predictor of treatment effect [26–28]. However, abstinence self-efficacy ratings are substantially affected by very recent experiences in abstaining [20, 29]. In the four sources of self-efficacy mentioned by Bandura [30], the most effective way of changing self-efficacy is through mastery experiences. Successes build a robust belief in one’s personal ability, while failures undermine it. A second way of creating self-efficacy is through the vicarious experiences provided by social models. Seeing people similar to oneself succeed raises observers’ self-efficacy. Social persuasion is a third way. People who are persuaded verbally that they are capable are likely to try hard enough to succeed than if their abilities are doubted. People also rely on their physical and emotional states to judge their abilities, for example, people judge fatigue and negative emotions as signs of inability [30].

### Other related theories and drug use

Besides self-efficacy, proposed by Bandura in his social learning theory, other researchers developed many theories related to drug use and abstinence. Here, we discuss two of them: Seligman’s learned helplessness theory and Weiner’s attribution theory.

Seligman suggested that inability to escape failure experiences may lead to learned helplessness [31, 32]. Bandura also wrote that unless people believe they can produce desired effects by their actions they have little incentive to act or to persevere in the face of difficulties [33]. In the case of drug users, because drugs are highly addictive, it is easy to relapse. If a person repeatedly fails to quit drugs, the person may “learn” to be helpless. However, besides underestimating one’s ability, Bandura wrote that overestimation or overconfidence in one’s ability may also lead to a negative outcome [34].

In 1978, Abramson, Seligman, and Teasdale presented a “critique and reformulation” of the learned helplessness model [35]. In the revised model, they suggested that causal attribution is a mediator in the process by which uncontrollable events produce helplessness.

In Weiner’s attribution theory, one dimension of attribution is internal/external [36]. People may have high self-efficacy if they attribute success to internal factors, such as ability, but attribute failure to external reasons, such as luck. However, people may have low self-efficacy if they attribute success to external reasons but attribute failure to internal reasons. Accordingly, drug users may remain to be high in self-efficacy if they attribute relapse(s) to external attributions; but they may have low self-efficacy or a sense of helplessness if they attribute relapse(s) internally to themselves.

### Current study

In this study, we firstly intended to explore the drug users’ abstinence self-efficacy in a compulsory drug detention center, an enforced and isolated place where little research has been undertaken, as well as their sources of abstinence self-efficacy.

We also intended to find out drug users’ attitudes towards compulsory drug detention. Although in many researchers’ views, compulsory drug detention is neither ethically acceptable nor an effective way to treat addiction [6, 7], attitudes from people who actually experienced it - drug users in compulsory drug detention centers - have never been studied.

We chose qualitative methods for two main reasons: 1) Because drug users’ terms of detention partly depend on their performances in drug detention centers, some addicts learn to disguise their real beliefs and thoughts. For example, some may disguise answers in quantitative studies according to what they think is going to be helpful to them. Qualitative research methods involve considerable time in building rapport with participants so that participants are likely to be more honest in their responses [37, 38]. 2) Qualitative methods are powerful for studying process [37, 38]; and have been shown to be very effective in many addiction studies [39].

## Methods

### Characteristics of the study location

Based on accessibility/convenience, we chose a male compulsory detention center in Shanghai to be the study location. The center had about 500 residents, located in an outer suburb of Shanghai and only accessible by buses belonging to the center. Although isolated, family and friends can visit, call, write mail or bring daily necessities. However, to ensure totally drug-free surroundings, everything brought in is checked by the police. As a consequence, we were not permitted to bring anything into or out of the center except a handbook, a pen, and a voice recorder.

The residents’ time was occupied with labor (e.g., sewing clothes) and educational classes (e.g., about drug laws), except for Wednesday mornings when they had free time. We chose that time to interview, so that interviews would not affect their performance evaluation. The research lasted more than half a year, from fall 2012 to spring 2013. Interviews were held in a closed meeting room, guarded by police to ensure our safety, but the police could not hear what we were talking about. The police agreed not to inquire into the details of interviews, and for the participants’ data to be anonymous.

### Data collection

Because of the special nature of a compulsory rehabilitation center, participants had to be selected by the police first to ensure our safety: only drug users who had completed the first stage - detoxification - were included. Nevertheless, to avoid a potential selection bias, we required the policemen who had no direct interactions with drug users to select participants. Three more participants were interviewed after the information was saturated, until no further information was added [40].

Each participant was interviewed about six categories of questions: (1) their history of drug use and rehabilitation, e.g., “how did you start to use drugs?”; (2) the reasons for their relapse(s), e.g., “would you tell me why did you re-use drugs?”; (3) their self-evaluation of addiction, e.g., “how do you perceive drug use?”; (4) their willingness, confidence and motivation to abstain, e.g., “do you want to abstain?”; (5) their plans and worries about their future, e.g., “what are your plans for the future?”; (6) their attitudes towards compulsory rehabilitation and other rehabilitation methods, e.g., “how do you perceive compulsory rehabilitation?”. To build rapport and obtain reliable information, before asking the above questions, we spent some time interviewing them about their childhood, family and other matters.

In total, 36 participants were included. Each participant was interviewed one to three times, and each interview lasted from 60 to150 min. Interviews were conducted by two graduate students well trained in qualitative research methods and familiar with the interview questions. They practised interviews together before the formal study. A participant was always interviewed by the same interviewer. Interviews were conducted in Chinese; and all audiotapes and notes were transcribed into Chinese in the evening immediately after a day’s interview.

### Data analysis

MAXQDA 12 was used for data management and analysis. During the study process, the research team (including two interviewers) had weekly meetings, to discuss the analysis and reflect on the process. All the results were translated into English. The team reached consensus on both the results and translations.

Firstly, the team read each transcript briefly and created case summaries. We coded and analyzed transcripts into concepts and themes, according to the grounded theory open coding method of thematic analysis [40, 41]. Analysis of each transcript was repeated until no new codes could be added.

Three themes were derived from our analysis: 1) characteristics of self-efficacy (including self-evaluation of addiction, perceived control, motivation for rehabilitation and causal attributions); 2) sources of self-efficacy (including mastery experience, vicarious experience, social support and persuasion, as well as psychological and physical factors); and 3) attitudes to compulsory drug detention.

We detected two different patterns in the theme “characteristics of self-efficacy”. If a participant underestimated the levels of addiction, overestimated his self-control, held no internal motivations and held external attributions, we classified the participant into an “overconfidence” group (*n* = 16). If a participant overestimated the levels of addiction, underestimated his self-control, held strong internal motivations and attributions, we classified the participant into a “helplessness” group (*n* = 17). Three participants could not be categorized into the two groups and we have classified these as “non-specific”.

We then compared the three groups’ sociodemographic characteristics, sources of self-efficacy and attitudes to compulsory drug detention.

## Results

To make the presentation of results clear, we first show the sociodemographic characteristics and the comparison between the overconfident group and the helpless one. Then we elaborate upon the characteristics of two opposite patterns of self-efficacy. We also show the differences between the two groups in sources of self-efficacy and attitudes to compulsory drug detention. After that, we elaborate upon the results of the “non-specific” group. Finally, we present a case to show how a drug user may change from “overconfidence” to “helplessness”.

Where quotations are used, the participants are identified by a number, followed by a group abbreviation (OC stands for “overconfidence”, HL stands for “helplessness”, NS stands for non-specific). We used heroin to represent opioid drugs and ice (methamphetamine) to represent synthetic drugs in this article, because that was how participants referred to them.

### Participants’ characteristics

Thirty-six participants were recruited. Their sociodemographic characteristics were extracted from both official records and interviews. They had an average age of 31.61 (*SD* = 5.34), ranging from age 20 to 43. The median years of drug use was 9.39 (*SD* = 4.71), ranging from two to 16. The median number of times of compulsory rehabilitation was 1.83 (*SD* = 1.00), ranging from one to four times (Table 1).

**Table 1:** Summary of participants’ characteristics

	All participants	Overconfidence	Helplessness
	(*N* = 36)	(*N* = 16)	(*N* = 17)
Age, *M (SD)*	31.61 (5.34)	30.50 (6.06)	33.12 (4.64)
Initial age of drug use, *M (SD)*	22.11 (4.86)	23.44 (4.79)	20.94 (4.88)
Year of drug use history, *M (SD)*	9.39 (4.71)	6.94 (4.89)	12.06 (3.27)
Times of compulsory detention, *M(SD)*	1.83 (1.00)	1.25 (0.45)	2.47 (1.07)
1st time, *N (%)*	18 (50.0)	12 (75.0)	4 (23.5)
2nd time, *N (%)*	9 (25.0)	4 (25.0)	4 (23.5)
3rd time, *N (%)*	6 (16.7)		6 (35.3)
4th time, *N (%)*	3 (8.3)		3 (17.6)
Drug type			
Heroin, *N (%)*	6 (16.7)	1 (6.3)	3 (17.65)
Ice, *N (%)*	14 (38.9)	9 (56.3)	4 (23.5)
Both, *N (%)*	16 (44.4)	6 (37.5)	10 (58.8)
Main method of drug-use			
Nasal, *N (%)*	7 (19.4)	5 (31.3)	1 (5.9)
Injection, *N (%)*	29 (80.6)	11 (68.8)	16 (94.1)
Marital status			
Single, *N (%)*	11 (30.6)	6 (37.5)	5 (26.4)
Girlfriend, *N (%)*	7 (19.4)	5 (31.3)	2 (11.8)
Married, *N (%)*	9 (25.0)	4 (25.0)	3 (17.6)
Divorced, *N (%)*	9 (25.0)	1 (6.3)	7 (41.2)
Number of children			
1 Child, *N (%)*	10 (27.8)	3 (18.8)	5 (29.4)
2 Children, *N (%)*	5 (13.9)	1 (6.3)	4 (23.5)

According to participants’ responses on the theme “characteristics of self-efficacy” below, we classified participants into three groups: 16 into the overconfident group (OC), 17 into the helpless group (HL) and three participants into the non-specific group (NS). It appeared that compared to OCs, HLs were older, had younger initiation age, longer years of drug use history and more sentences to compulsory drug detention; more HLs used heroin or heroin and ice at the same time, and mainly by injection; more HLs were divorced but with more children.

Here, overconfidence is referred to as the overestimation of one’s ability to abstain from drug use and helplessness as the underestimation of it. Participants who were overconfident or helpless showed different patterns across the four sub-themes elaborated below: self-evaluation, motivation for rehabilitation, perceived control, and causal attribution.

### Self-evaluation: “I am not addicted” versus “I am too addicted”

From the data we identified two self-evaluation categories: perceptions about drug use and evaluations about addiction.

- Perceptions about drug use
  As to perceptions about drug use, OCs reported a) they were not using drugs, “*I still think it’s just a kind of dope, not drugs”* (#18, OC); b) using drugs was normal, “*You know, 80 % white-collars in Shanghai use drugs”* (#22, OC); or even c) drug use was helpful “*Drugs accompanied me”* (#26, OC).
  On the contrary, HLs realized the harm of drugs. They claimed that taking drugs wasted their time (“*I had drugs for more than 10 years, I wasted more than 10 years, it’s meaningless”*, #7, HL) and money (“*Drug is not a good thing, and cost me a lot of money”*, #17, HL), destroyed their health (“*I got hepatitis because of using drugs”*, #15, HL) and devastated their spirit (“*I felt sleepy all day”*, #3, HL).
- Evaluations about addiction
  OCs held a belief that “*I am not addicted*”. They stated this belief mainly by expressing a low frequency of drug utilization (“*I use drugs about ten times a year, it can’t be called addicted.”,* #6, OC) or by denying a reaction to abstinence (“*I’m not very addicted to drugs, I might think about it sometimes, but if there’s no chance to use [drugs], I feel OK.”,* #14, OC).
  In contrast HLs said that “*I’m too addicted*” and might never be able to get rid of drugs. They lost their confidence and hope for their futures.
  But two claims were common for both OCs and HLs. One was that physical addiction was easier to get rid of than psychological addiction; the other one was that the abstinence reaction of heroin was stronger than that of ice. So to some extent, participants who used heroin as drugs were more likely to admit their addiction. This is consistent with the results mentioned above that more HLs used heroin or heroin and ice at the same time than OCs.

### Perceived control: “I can control” versus “I can’t control”

From the data on perceptions of control, two categories emerged: perceived control over drug use and confidence in rehabilitation.

- Perceived control over drug use
  OCs did not only claim that they were not addicted, they also did not take drug addiction seriously. Some participants stated that drug use was all under their control. The most frequently used phrase was that “*I use drugs when I want, and don’t when I don’t.”* (#31, OC).
  However, it may be because OCs thought they could control their use of drugs, they would not reject the invitations from old drug friends, and they would easily give in to the temptation to re-use.
  The thing HLs worried about most was drug friends. Here, the worry about drug friends reflected their worry about relapse. They stated that they could not control drug use, especially by themselves:
  *“If someone’s governing you, you will have stronger will power. If nothing could restrict you, you seem could not control yourself…I know I cannot control myself…most important is the contacts with old friends, there are lots of things outside that you cannot avoid…”(#1, HL).*
- Confidence in rehabilitation
  OCs stated that they have faith in controlling themselves to not touch drugs.
  *“I believe that I have the willpower to stay away from it, I know I won’t touch it again.” (#14, OC).*
  In contrast, many HLs worried about reusing drugs, showing a feeling of uncertainty and lack of confidence.
  *“I know I could get rid of drugs here, and I’m certain I won’t touch it for a while after that… but I’m not sure about the future… maybe later I will think about drugs and get back to it.” (#7, HL).*
  However, a common point made by OCs and HLs was that they would not guarantee that they would not use drugs again. For HLs, it was because of their lack of confidence, but for OCs, it was because of their lack of motivation.

### Motivation to rehabilitate: “There’s no need” versus “I really want to”

This sub-theme was categorized into amotivation, extrinsic motivation and intrinsic motivation.

- Amotivation
  Amotivation seemed to apply to a number of OCs but not to any HL participants. When asked about the future, many OCs said they did not have any worries, they just “*let it go”* (#8, OC). They said there was no need or necessity to rehabilitate from drug use: “*There’s no need to rehab, I’m not addicted. I don’t know why I’m forced to [rehab].”* (#18, OC).
  Several OCs even directly said that they will re-use drugs hereafter. For example, #27 said he will re-use drugs because: “*the more they [parents] say not to, the more I want to use.”* (#27, OC). And #8 was disgruntled about compulsory drug detention for wasting two-years of his time.
  *“People are antagonistic…my mother wanted me to get married in 3 years, now 2 years in here, how could I get married in 3 years?…I wasted 2 years here, I think if I did not use drugs when I’m out, then what is the meaning of the 2 years?…Normally, I should not use again, but I feel antagonistic…” (#8, OC).*
- Extrinsic/intrinsic motivations
  Some OCs did have extrinsic motivations towards rehabilitation. The reason was time pressure (“*I’m older and older*” (#36, OC)), money pressure (“*When out of money, I want to abstain*” (#14, OC)), stigma (“*I thought if I could abstain, fewer people will know I had a history like that.”* (#29, OC)), the two-year cost of compulsory drug detention (“*If I knew that I would pay for 2 years, I wouldn’t have used it. The cost is too big.”* (#32, OC)), and family members (*“Not for others, for my parents and my daughter, I had to abstain.”* (#6, OC)).
  More HLs reported these external motivations for rehabilitation. Also, many of them showed strong internal motivations.
  *“Actually I deeply wanted to abstain, drug users all wanted to abstain.” (#12, HL)*
  In addition, we could see this from their behavior. They took numerous methods to abstain, such as methadone treatment, locking themselves up, isolating themselves or going to voluntary rehabilitation centers on their own. But OCs seldom adopted these actions, and only if they had been forced to by their family members.

### Causal attribution: “It was not my fault” versus “I deserved it”

Here we identified two categories: drug users’ attributions for being forced to take compulsory drug detention and their attributions for poor consequences along with it, such as divorce, deprivation of freedom and cost of time. We further divided attributions into internal or external.

- Attributions for taking compulsory drug detention
  Many OCs claimed “*it’s not my fault*”. They attributed externally and did not want to take responsibility. Some found excuses for being arrested.
  *“I was caught when getting drugs this time, but it was not for myself, it was my friend who called to me, he said he needed some and asked me to get drugs for him.” (#5, OC).*
  Many OCs blamed the drug policy system. They said the policy was unfair. They expressed it directly, or indirectly by using the word “*only*” and “*seldom”* or by asking “*why is it me?*”
  *“I seldom use drugs, in the past year I only used drugs for a time and I got caught… It’s unfair, only one time…” (#18, OC).*
  *“In other countries, it’s legal to use drugs, here we have to be deprived of freedom. Do you think it’s fair?” (#6, OC).*
  In contrast, HLs attributed internally; they blamed themselves for using drugs. They admitted it was their fault. They started to take the responsibility.
  *“I know what I did was wrong, I should take responsibility for what I have done.” (#34, HL)*
- Attributions for negative consequences
  Almost all OCs attributed externally and blamed compulsory rehabilitation for poor consequences. They said their stable life has been destroyed by it.
  *“They said I’m lucky because for 16 years, this is my first time being caught. But I do NOT think so. I’m unlucky to be here, I couldn’t work and make money, I paid a lot.” (#8, OC).*
  HLs attributed internally. In HLs’ interviews, many topics were about confessions, guilt and regrets. They claimed that they deserved those negative consequences.
  *“I hate myself. I know I disappointed my family, my wife left me. I think she should have left me; she deserves a better man.” (#21, HL)*

### Sources of self-efficacy

#### Mastery experience

- Direct experience with rehabilitation
  As we have seen in Table 1, HLs had longer years of drug use, and more times of compulsory detention than OCs. However, there were exceptions. For example, #9 (HL) only had a drug use history of 5 years and this was his first compulsory rehabilitation, but he already lost confidence. He once took a methadone treatment, first pressured by his girlfriend, then motivated by himself. The treatment lasted for 2 years but failed. It was this two-year failure experience that reduced his self-efficacy.
  In contrast, #10 (OC) used drugs for 16 years and this was his second compulsory rehabilitation, but he showed obvious “overconfidence” because of his successful experiences of abstaining from heroin for 2 years.
  As a result, successful and failure mastery experiences with rehabilitation (not just limited to experiences with compulsory detention) could both change a person.
- Interpretation of experiences
  How the drug users interpreted their experiences might also be crucial to their self-efficacy. As for #10 (OC), he interpreted the two-year rehabilitation as a success experience. His focus was that “*I abstained from heroin*”, but neglected the fact that he turned into using ice thereafter. In contrast, the same experience was interpreted as a failure by HLs. For example, #3 (HL) did not use drugs for four years after the second compulsory detention, but his focus was that he re-used drugs, but neglected the fact that he managed to control himself for four years. This “interpretation bias” also showed in other OCs and HLs.
- Attribution of relapse
  Attribution of relapse was also different between OCs and HLs. Several OCs, when talking about relapse, attributed it to external reasons. For example, participant #36 (OC) once locked himself up to rehabilitate, but after 3 days, he gave up, re-connected with his old friends and re-used drugs. He said:
  *“Why I went out, it’s not because I need drugs or I can’t hold it, it’s because of something in my business, I had to go outside” (#36, OC).*
  However, HLs often attributed relapse to internal reasons. They thought relapse re-confirmed that they could not control themselves and they could not rehabilitate.

#### Vicarious experience

Both OCs and HLs had no successful models who never used drugs again (except #1 (HL), which we will explain below), but OCs were not as affected by failure models as HLs. As we stated before, OCs did not have strong motivations to rehabilitate and some of them said drug use was good or normal. Consequently, failure models who relapsed were not “models” in the perspectives of OCs. Their models were people who were using drugs, such as the “80 % white-collars in Shanghai” reported by #22 (OC). Here, “white-collars in Shanghai” were representatives of people of high status. Thus, #22 (OC) expressed that drug use was not a bad thing, rather, it was a thing that belongs to people of high status.

In contrast, HLs were afraid they may end up as failure models. For example, #16 (HL) knew people who had died from drug use and he was afraid he may die that way someday. Participant #4’s (HL) sister started using drugs earlier than he did but she did not stop drug use until recently; he was worried that he was like his sister - unable to abstain.

Participant #1 (HL) was the only person who said he had a friend who successfully abstained from drugs and never used again (at least until the time of our interview). In order to rehabilitate, his friend went to Tibet for two years, with no money. However, #1 (HL) said it was too hard for him to do that. It seemed that the successful model was not considered to be a model that he could learn from.

#### Family support and persuasion

Here, we discuss persuasion drug users received from their family members.

Persuasions OCs received could be divided to three types: powerless persuasion, mild persuasion, and encouragement. In powerless persuasion, no matter what other people said, the drug users would not listen. In their words, the family members were unable to “*guan*” (take control of) them. In mild persuasion, the family members were trying to persuade them to get rid of drugs with patience, by saying things like “*using drugs is not good*”, “*be good here [the center]*” and so on. In encouragement, the family members showed their confidence in drug users. For example, participant #14’s (OC) wife said to him:

*“You can abstain, we need you at home, and we need you to take the responsibility as a man.” (#14, OC).*

Persuasions HLs received were far different from those received by OCs. The most prevalent type was blame. The family members were angry at them, no matter before compulsory detention or when they visited drug users during that time, they quarreled with and blamed drug users. Examples of blame sentences used frequently were: “*how could you…*” and “*people like you…*” By saying these, the family members were not just blaming drug users for having done something bad, but also suggesting that they were bad people.

HLs also experienced a lack of attempts at persuasion, where the family members gave up on drug users. Some HLs divorced their wives; some broke up with their girlfriends; and several HLs said their parents became numb and did not “*guan*” (take control of) them anymore (this was different from OCs’ “unable to *guan*”, in which family members tried to “*guan*”, but could not control OCs; here, HLs’ family did not try to “*guan*” them).

However, family members also motivated HLs to abstain, they did not want to disappoint family members again, but also afraid that if they could not abstain this time, they would be abandoned.

*“Even though they [parents] yelled at me, they would come to visit me. My mother went to hospitals several times because of me…I thought I would rather die than using drugs [before this time’s compulsory drug detention]…[Now] I would think a lot, afraid that I could not abstain, could not control when outside, and my wife would actually divorce me…” (#15, HL)*

#### Psychological and physical factors

OCs reported less negative emotions and better physical status than HLs. Although some OCs reported anger towards the compulsory detention center, none of them showed any signs of depression. However, many HLs reported that they felt low in mood, unhappy and lost. They did not know what to do, and did not want do anything. If they needed to do something, they felt spiritless and became tired easily. Several of them cried during the interview and several even said they would rather die.

### Attitudes to compulsory rehabilitation: “It’s not useful” versus “It’s the most effective way”

Here, we report drug users’ attitudes to compulsory rehabilitation, especially whether they thought it was useful or not.

OCs held an opinion that it was not useful. Actually, according to them, no way was useful, except one’s own will power or motivation.

*“I cannot tell whether it’s useful, the most useful way is whether you want to rehab or not, if you are determined to rehab then you will, other ways cannot help.” (#13, OC)*

In contrast, HLs claimed compulsory drug detention to be a good, even the best way. When asked about the effectiveness of compulsory drug rehabilitation, many HLs said: “*it is the most effective way*”*.*

It was a conclusion they arrived at after their numerous methods to abstain. For example, this was #33’s third time. He attempted many methods to abstain over time. But he would take drugs at the same time as his methadone therapy; he came out only a few days after entering the voluntary rehabilitation center and he went to another province, which is more than 1000 miles away from his home, but half a month later, he could not resist the temptation to use drugs and he came back home to re-use. He also asked his wife to tie him to the bed, but after seeing his pain she became softhearted and untied him. He said:

*“These methods couldn’t last for a long time. I have a weak will. Only in the compulsory rehabilitation center could I really get rid of drugs for two years.” (#33, HL)*

### Non-specific group

Three participants were classified into the non-specific group. Below, we report their self-efficacy characteristics, sources of self-efficacy and attitudes to compulsory drug detention.

The three non-specific participants (#2, #20 and #28) all admitted that they were addicted, even though #2 said he was not very addicted. However, unlike HLs, #28 said he believed he could control himself; #2 and #20 had worries about the relapse but still had confidence in themselves. At the same time, unlike OCs, they had internal motivations to rehabilitate in addition to external motivations. #20 and #28 attributed poor consequences along with drug use and compulsory drug detention to themselves and drug use, while #2 blamed compulsory detention. In conclusion, non-specific participants admitted addiction, had motivation to rehabilitate, perceived a sense of control and had internal attributions. It seemed that they had high self-efficacy but this high self-efficacy did not lead them to become like OCs that lack motivation to change.

This was #2 and #20’s first time of compulsory detention and #28’s second time, but they had all tried other ways to rehabilitate. However, these failure experiences did not affect their sense of control, rather, as #20 said: “*I learned from these experiences, now I’m better able to control*”. They all did not have success models, and that is one thing they worried about; but they also had a belief that they were not like their drug friends and they could rehabilitate. The ex-wife of #28 used divorce as a threat but kept visiting him every month and encouraged him that once he rehabilitated, they would re-marry. This gave #28 motivation to rehabilitate. However, the family of #2 said they were humiliated by him and the family of #20 blamed him for using drugs. The psychological and physical status of #2 and #20 were also different from #28, they experienced a sense of spiritlessness and fatigue, and worried about the future; while #28 did not.

As for attitudes to compulsory drug detention, #28 said it was necessary; and #2 and #20 said it was useful. Although #2 blamed compulsory detention, he also said that mild persuasion was not enough and not working, drugs were so hard to rehabilitate that strong external control methods like compulsory detention were needed.

### Transition from “overconfidence” to “helplessness”

Here, we present a case to show how a drug user may change from “overconfidence” to “helplessness”, but we are not suggesting that “overconfidence” and “helplessness” are two stages of drug use experience. There are possibilities that not all OCs would turn into “helplessness” eventually, nor were all HLs turned into “helplessness” from “overconfidence”.

Participant #24 (HL) described his transition from the first time of compulsory drug detention to his present third time in detail. He talked about his drug initiation. At first, he had not realized its harm. Then he was caught by anti-drug police and he was only required to take community drug detention. But several months after community drug detention, he was caught again. And this time, he was sent to compulsory drug detention. At that time, like OCs in our interviews, he felt it was unfair and blamed compulsory drug detention.

*“I hated here, I thought I wasn’t addicted, why you caught me here…I was young and I didn’t realize something. In my eyes, at that time, drug was no big deal, it’s like, eating…” (#24, HL).*

When he was out, he soon went back into the old friends’ circle and re-used drugs. Then he was arrested and sent for the second compulsory drug detention. He talked about his changes:

*“…maybe because I was older, and I saw more things, mostly, I saw some of my friends who bought cars and houses, but I had nothing. I was touched. I wanted to rehab…I got divorced and I’m a father now…but I didn’t have stronger will power, I couldn’t resist the temptation, I re-used [drugs] again…” (#24, HL).*

It was external reasons that made him want to rehabilitate, but he failed and re-used drugs after the second time. During a long period between the second and third time, he tried numerous ways to rehabilitate by himself. Some only lasted for several days, some lasted for several months. But all failed. At the time of our interview, it was his third time:

*“I don’t know what it will be like in the future, I mean, I want to rehab, I really want. But I know I can rehab here, but I don’t have the faith when I’m out…if I knew it [drugs] was so hard [to rehabilitate], I would never have tried it, I would never have used it…” (#24, HL).*

## Discussion

Previous researchers found that heroin dependent drug users in compulsory rehabilitation centers in China lacked the motivation to change and were low in self-efficacy [42]. However, our research found that male drug users were not all low in motivation and self-efficacy—some of them were low in motivation but high in self-efficacy, while some of them were high in motivation but low in self-efficacy.

Usually, self-efficacy has been considered to be positively related to performance. However, Vancouver et al. found a positive relationship between self-efficacy and performance may partially be a function of performance’s influence on self-efficacy, but not the other way around [43]. Instead, high self-efficacy may lead to overconfidence in one’s ability [43]. Stone found people with high self-efficacy were less motivated and contributed less in tasks [44]. This was consistent with our findings that some drug users were high in self-efficacy but lacked motivation to abstain. We categorized this kind of drug user into the “overconfidence” group (OCs). They seemed to be in the first stages of the Transtheoretical Model and the Precaution Adoption Process Model [45, 46], that is, unaware of the risk of drug use, unengaged in changing behaviors or undecided about changes to their conduct. This does not augur well for their recovery, because a prerequisite to beginning recovery is the admission of addiction and personal powerlessness [47].

In contrast to OCs, HLs had low self-efficacy. Self-efficacy was substantially affected by success or failure experiences in abstaining [29]. Our research is consistent with those findings: HLs had experienced more years of drug use and more periods of compulsory rehabilitation, indicating that they had more relapses. According to learned helplessness theory, their sense of helplessness and low self-efficacy might be learned from those relapses [31]. Learned helplessness may also lead to depression; and HLs in our study showed negative emotions and poor physical status, such as low mood, fatigue and a feeling of loss [31]. Yet, Seligman revised his theory and proposed that helplessness and depression comes from negative attributions [31]. HLs in the research regarded relapse as a failure, and attributed failure to themselves. It may be the self-attribution, not just relapse, that lead them to the sense of losing control and then increase the probability of relapse. This establishes a vicious cycle and they “learned” to be helpless.

According to the results and the revised model, we suggest treating the two kinds of drug users differently. For OCs, in the first place, we need to increase their motivation to rehabilitate by assisting them to realize the harm of drug use, and helping them admit the addiction and personal powerlessness. Drug education programs may be a good choice. Social support treatment may also be helpful. Research has indicated that social support treatment was more effective than self-control treatment in high self-efficacy participants [48].

However, to HLs, self-control treatments could be more effective. One reason may be that they lacked social support. Some of them have already been abandoned by their family members. But family members also motivated them to abstain. So if we could work with their family, it may be effective if we could teach them ways to communicate with drug users, such as how to encourage HLs instead of blaming and yelling at them.

In particular, we need to help HLs increase their sense of perceived control. One way is to change their interpretations of experiences. Interpretation of the same experiences was different between OCs and HLs. Re-using drugs after 4 months of abstinence may be interpreted as a success to OCs, but as a failure to HLs. As a result, to increase the self-efficacy of HLs, we need to adjust their goal expectancy. They could start by setting smaller goals, such as not touching drugs for a month. Then every time they reached a goal, give them a reward. Even though they may re-use drugs, helping them focus on the fact that they managed to not touch drugs for a certain time and this was a success, next time they could try to keep off drugs for a longer time.

Meanwhile, mental health programs or skills-training techniques have also proved to be helpful [49, 50]. Although the Chinese government required every compulsory drug detention center to have special counsellors, the number and qualifications are not sufficient [17], suggesting adjustments and improvements are needed.

Importantly, we consider HLs felt helpless without compulsory drug detention, but the feeling of helplessness did not generalize to every area [51]. Instead, they accepted the effectiveness of compulsory drug detention. Compulsory drug detention, to some extent, works as a strong external support for them. Some western researchers now back compulsory rehabilitation for young addicts [52]. As  the author said:

*“There’s many young people who have a really strong will to do something about their substance abuse but they just can’t stay in treatment in a voluntary capacity…their dependency is too powerful”* [52]*.*

However, compulsory drug detention is like a double-edged sword. On the one hand, its supportive role to some drug users should not be ignored. On the other hand, it makes them feel that they could not stay away from drugs once outside of the compulsory drug detention center. Without a mandatory method, they have low confidence and self-efficacy. Accordingly, HLs entered into a vicious cycle: compulsory drug rehabilitation - relapse - compulsory drug rehabilitation- relapse… “*It’s not hard to get people off drugs, it’s really hard to keep them off drugs.”* [52]. In particular, how to keep them off drugs outside of compulsory drug detention should be the focus of future work and drug control policies in China. Transitional recovery programs may be helpful [53, 54]. That is why the Chinese government required drug users to take community recovery after two-years compulsory detention. However, during community recovery, drug users had little supervision and received little professional treatment. Drug users had to depend on themselves. Thus, although community recovery tends to build a bridge between external control and internal control, the goal seems not to have been achieved.

At the same time, the policy of compulsory detention suggested that if a drug user was found re-using drugs after the first-time of compulsory detention, he or she will be forced to take the second-time compulsory detention, regardless of the drug-using frequency and how long he or she managed not to touch drugs. This policy will reinforce HLs’ interpretation that “*as long as I re-used drugs, I failed*”; thus leading to HLs’ feelings of guilt and shame, as well as to low self-efficacy.

Overall, unlike some researchers’ calls for closure of compulsory drug detention centers [6, 7], we think neither only compulsory drug detention nor removal of it is advisable. Compulsory drug detention does have strengths in supporting people who feel helpless, although it also has many weaknesses.

Moreover, as we stated above, HLs had longer years of drug use and more periods of compulsory drug detention than OCs, suggesting they had more relapses. Our results also revealed that HLs were older; more of them used heroin mainly by injection and were divorced and had more children.

HLs had higher motivation to rehabilitate than OCs, one reason may be they were older and experienced more time pressure. Another reason may be even though more of them divorced, HLs had more children who were a major motivation for them to abstain. The third reason may be more HLs used heroin but heroin induces a heavier abstinence reaction than ice, and it may be easier for drug users who took heroin to realize the addiction. Also, almost all HLs turned to injection to replace nasal use; and injection leaves marks that may “help” to realize addiction. However, HLs with longer years of drug use may also be older, easier to get divorced, had more children, started with using heroin because heroin is an older drug and turned to injection to replace nasal use. As for marital status, although divorce may lead to worse treatment effects and then lower self-efficacy, lower self-efficacy may also lead to longer years of drug use and then divorce. In conclusion, it is hard to infer causal relationships between sociodemographic characteristics and self-efficacy.

### Limitations

Firstly, the research was conducted in a special setting, where participants had to be selected by the policemen. Even though we asked policemen who had no direct interactions with drug users to select, potential selection bias could not be avoided completely. Secondly, participants in a compulsory environment might also have some concerns about telling the truth. To ensure the reliability of information, we began with a number of warm-up questions to build rapport. These two major limitations are realistic problems any researchers may face, especially those who try to do research in a special place such as an isolated compulsory drug detention center.

The study had some other limitations. For example, because we did not follow drug users’ self-efficacy over a prolonged period, the dynamic transitional process between “overconfidence” and “helplessness” could not be fully understood. In addition to longitudinal and quantitative studies, research that recruits female participants, and research undertaken in other countries or other places in China are needed.

## Conclusion

Two opposite types of self-efficacy of male drug users in a mandatory drug rehabilitation center were found: “overconfidence” and “helplessness”. Overconfident participants (OCs) underestimated addiction, overestimated self-control, held external motivations and attributions. In contrast, helpless participants (HLs) overestimated addiction, underestimated self-control, and held internal motivations and attributions.

Group comparisons found HLs had longer years of drug use and more relapse history than OCs. HLs were also more inclined to interpret relapse as a failure and attribute relapse to themselves. While both OCs and HLs had failure models, they lacked successful models, but this did not affect OCs because they did not regard failure models as “models”. Compared to OCs, more HLs were abandoned by their family members and received more blame from family members instead of encouragement, but family members motivated them to abstain. HLs also experienced more negative emotions and had worse physical status.

A combination of self-efficacy theory, learned helplessness theory and attribution theory was used to explain the results. For OCs, the most important thing was to increase their motivation; while for HLs, it was important to increase their perceived control by changing their interpretation of relapse experiences.

The two different groups also showed distinct attitudes to compulsory drug detention, OCs were opposed to it, while HLs approved of it. Compulsory drug detention might serve as a strong support to drug users who are in our “helplessness” category but more effective treatments and qualified special counsellors were needed. In addition, how to keep drug users off drugs outside of compulsory drug detention should be the focus of future work and drug control policies in China.

In conclusion, the study highlighted the characteristics of two opposite types of abstinence self-efficacy of drug users, explained the possible reasons of their differences, and pointed out the supportive role of compulsory drug detention. The study also gave suggestions for the improvements of compulsory drug detention and the treatments of drug users.

## Additional files

Additional file 1: Interview guidelines. (DOCX 14.6 kb) Additional file 2: Information of each participant. (XLSX 25.6 kb)

## Abbreviations

CD: Compulsory drug detention
HL: Helplessness
NS: Non-specific
OC: Overconfidence
