# Supplementary material for: “Overconfidence” versus “helplessness”: A qualitative study on abstinence self-efficacy of drug users in a male compulsory drug detention center in China
Source: Subst Abuse Treat Prev Policy. 2016 Aug 31;11(1):29. doi: 10.1186/s13011-016-0073-2 (PMC5006575; doi:10.1186/s13011-016-0073-2)
Supplement: Additional file 1: — Interview guidelines. (DOCX 14.6 kb) [file 13011_2016_73_MOESM1_ESM.docx]

**Interview guidelines**

**Part 1. History of drug use**

1. When did you start using drugs? What kind of drugs did you use at first? Have you taken any other drugs? (Why changed?)

2. How did you start to use drugs? What happened at that time?

**Part 2. History of rehabilitation**

1. How many times have you been to the compulsory drug detention center? Have you tried any other ways to rehabilitation? Would you tell me more about that?

2. Would you tell me why did you re-use drugs? What happened at that time? After re-using drug, what did you think? How did you feel?

3. Have you ever tried any ways to abstain drugs by yourself? (if yes) What did you do? Do you think is effective or not, why?

**Part 3. Self-evaluation of addiction**

1. How do you perceive drug use? What’s the meaning of drug use to you?

2. What’s others’ reaction to you using drugs? What’s their understanding of drug use?

3. What’s your understanding of addiction? Do you think yourself addicted? Why or why not?

**Part 4. Motivations to abstain**

1. Did you want to abstain drugs before here? Do you want to abstain drugs now? Why or why not?

2. Do you think you can control drugs? Why or why not?

3. What or who is your motivation to abstain?

**Part 5. Plans for the future**

1. What are your plans for the future? What about drug use?

2. Do you have any worries for the future? What are they? What’s the biggest worry?

**Part 6. Attitudes towards rehabilitation**

1. Among all the ways you had for rehabilitation, which do you think is the most effective? Which is the least effective? Why?

2. How do you perceive compulsory rehabilitation? What are the good and bad points of it?
